# Child survival: a message of hope but a call for renewed commitment in UNICEF report

**DOI:** 10.1186/1742-4755-10-64

**Published:** 2013-12-11

**Authors:** Tessa Wardlaw, Danzhen You, Holly Newby, David Anthony, Mickey Chopra

**Affiliations:** 1 Data and Analytics Section, Division of Policy and Strategy, United Nations Children’s Fund (UNICEF), 3 United Nations Plaza, New York, NY 10017, USA; 2 Social Inclusion, Policy and Budgeting Section, Division of Policy and Strategy, United Nations Children’s Fund (UNICEF), 3 United Nations Plaza, New York, NY 10017, USA; 3 Health Section, Programme Division, United Nations Children’s Fund (UNICEF), 3 United Nations Plaza, New York, NY 10017, USA

**Keywords:** Child mortality, Under-five mortality, Neonatal mortality, A Promise Renewed

## Abstract

A recent UNICEF report *Committing to Child Survival: A Promise Renewed Progress Report 2013* presents a comprehensive analysis of levels and trends in child mortality and progress towards MDG 4. The global under-five mortality rate has been cut nearly in half (47%) since 1990. However, during this same period, 216 million children are estimated to have died before their fifth birthday. Most of these deaths were from leading infectious diseases such as pneumonia, diarrhoea or malaria, or were caused by preventable neonatal causes such as those related to intra-partum complications. The highest mortality rates in the world are observed in low-income countries in sub-Saharan Africa and South Asia. Sub-Saharan Africa faces a particular challenge in that it not only has the highest under-five mortality in the world but it also has the fastest population growth. Progress is possible, however, and sharp reductions in child mortality have been observed at all levels of national income and in all regions. Some of the world’s poorest countries in terms of national income have made the strongest gains in child survival. Within countries, new analysis suggests that disparities in under-five mortality between the richest and the poorest households have declined in most regions of the world, with the exception of Sub-Saharan Africa. Furthermore, under-five mortality rates have fallen even among the poorest households in all regions. The report highlights the growing importance of neonatal deaths; roughly 44% of global under-five deaths — now 2.9 million a year — occur during the neonatal period, with up to 50% dying during their first day of life and yet over two-thirds of these deaths are preventable without intensive care. The report stresses how a continuum of care approach across the whole life cycle is the most powerful way of understanding and accelerating further progress.

## 

Global progress in reducing child deaths has been enormous, and yet the world is in danger of falling short on its promise to children to reduce the under-five mortality rate by two-thirds between 1990 and 2015 (Millennium Development Goal 4). A recent UNICEF report *Committing to Child Survival: A Promise Renewed Progress Report 2013*[1] presents a comprehensive analysis of levels and trends in child mortality and progress towards MDG 4, using data generated by the UN Inter-agency Group for Child Mortality Estimation [2]. The report’s findings highlight both the impressive progress in child survival over the past decade, as well as the work that remains to end preventable child deaths. In particular it highlights the growing importance of neonatal deaths (i.e., deaths that occur in the first twenty eight days of life); roughly 44% of global under-five deaths — now 2.9 million a year — occur during the neonatal period, with up to 50% dying during their first day of life and yet over two-thirds of these deaths are preventable without intensive care. The report stresses how a continuum of care approach across the whole life cycle is the most powerful way of understanding and accelerating further progress.

The global under-five mortality rate has been cut nearly in half (47%), from 90 deaths per 1,000 live births in 1990 to 48 per 1,000 in 2012. All regions in the world except Sub-Saharan Africa have achieved reductions of 50% or more, with three – East Asia and Pacific, Latin America and the Caribbean, and Central and Eastern Europe and the Commonwealth of Independent States—lowering their under-five mortality rates by 60% or more. The estimated annual number of under-five deaths worldwide has fallen from 12.6 million to 6.6 million over the same period, resulting in an estimated 90 million lives saved over the past 22 years (Figure 1).

**Figure 1:**
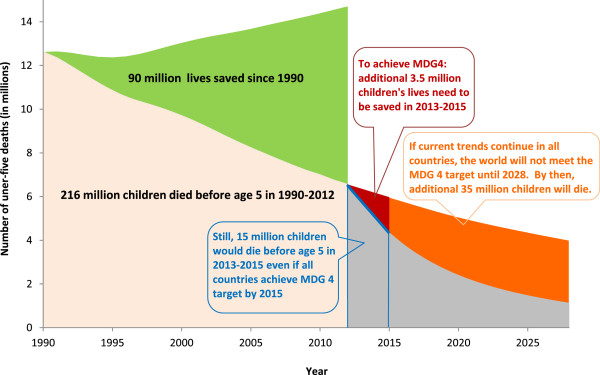
**Declines in under-five mortality have saved millions of lives worldwide.** Number of under-five deaths, 1990–2012 and projected 2013–2028.

However, during this same period, 216 million children are estimated to have died before their fifth birthday. This is more than the current total population of Brazil, the world’s fifth most populous country. In 2012 alone, around 6.6 million children died before their fifth birthday, approximately 18,000 per day. Most of these deaths were from leading infectious diseases such as pneumonia, diarrhoea or malaria, or were caused by preventable neonatal causes such as those related to intra-partum complications. And many under-five deaths occurred in children already weakened by undernutrition –a contributing factor in around half of global under-five deaths. Malnutrition and infectious diseases occur predominantly among the poor and their distribution is highly concentrated in low income countries. The highest mortality rates in the world are observed in low-income countries in sub-Saharan Africa and South Asia. Sub-Saharan Africa faces a particular challenge in that it not only has the highest under-five mortality in the world but it also has the fastest population growth. By 2050, this region is expected to have 37% of all under five children in the world. Unless the rate of decline of the under-five mortality rate outpaces the rate of population growth, the number of under-five deaths will increase.

Progress is possible, however, and sharp reductions in child mortality have been observed at all levels of national income and in all regions. Some of the world’s poorest countries in terms of national income have made the strongest gains in child survival (Figure 2). Seven high-mortality countries, six of them low income and one lower-middle income (Bangladesh, Ethiopia, Liberia, Malawi, Nepal, Timor-Leste and United Republic of Tanzania), have already reduced their under-five mortality rates by two-thirds or more since 1990. Many middle-income countries have also made tremendous progress in reducing under-five deaths, and most high-income countries have also seen sharp declines since 1990 — proving that even in high income countries, rapid declines in child mortality are possible. Within countries, new analysis suggests that disparities in under-five mortality between the richest and the poorest households have declined in most regions of the world, with the exception of Sub-Saharan Africa. Furthermore, under-five mortality rates have fallen even among the poorest households in all regions.

**Figure 2:**
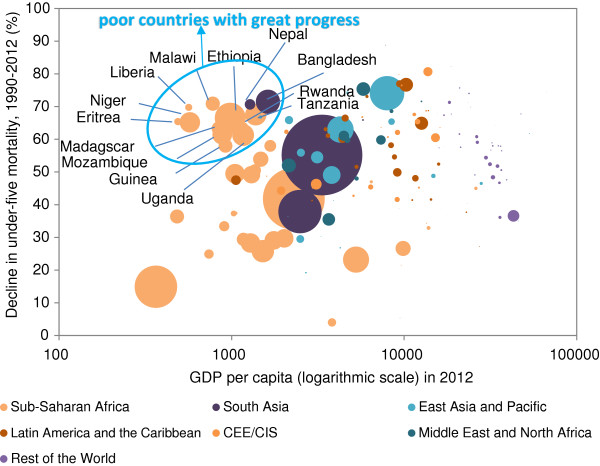
**Many low income countries have made significant progress in reducing child deaths.** Decline in under-five mortality rate, 1990–2012 and GDP per capita in 2012.

Children who die before they complete 28 days of life often do so as a result of diseases and conditions that are readily preventable or treatable with proven, cost-effective interventions. Globally, more than 20% of neonatal deaths were caused by sepsis and meningitis (12%) and pneumonia (10%) in 2012. Sepsis, meningitis and pneumonia are highly treatable, provided simple interventions and basic treatment knowledge are available. Another 34% of neonatal deaths, the majority of them preventable, were caused by preterm birth complications. Investment in maternal care, specifically labour and delivery care and other high-impact interventions focused on the 24 hours around the time of birth, holds the greatest potential for reducing neonatal mortality. Despite the increase in institutional deliveries globally since 2000, far too many births — in some countries, more than half — occur outside health facilities.

Recent success in reducing child mortality has been due to more effective and affordable treatments, innovative ways of delivering critical interventions to poor and excluded populations, and sustained political commitment. However the Report also draws attention to the broader social determinants of child mortality including early child marriage, which is common in many high mortality countries. Early marriage raises the likelihood of early childbearing (Figure 3), partly as a result of societal norms that influence when child-bearing is considered acceptable, or is even expected.

**Figure 3:**
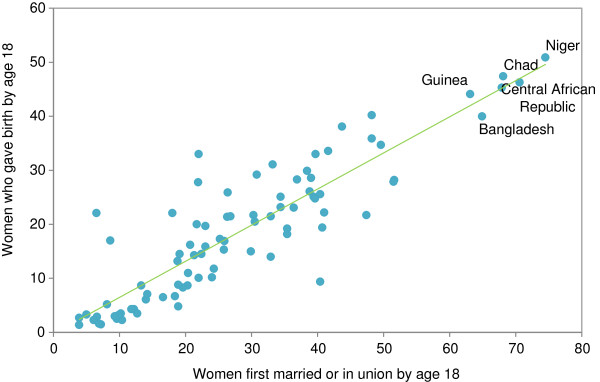
**Countries with high levels of child marriage tend to have high levels of early child bearing. **% of women aged 20–24 who were first married or in union by age 18 and % of women aged 20–24 who gave birth by age 18, in selected countries with available data, 2005–2011.

Without faster progress in reducing preventable diseases, the world will not meet its child survival goal (MDG 4) until 2028 — 13 years after the deadline — and 35 million children will die between 2015 and 2028 who would otherwise have lived had we met the goal on time.

This report shows that with political commitment, strong determination and dedication from all actors involved in child health, many deaths can be prevented and MDG4 can be achieved in low-and-middle income countries. A number of successful countries are leading the way to achieve this goal. By following their example, we urge all global health actors to commit to achieving a more fair and equitable world for all children.

## Competing interest

The authors declare that they have no competing interests.

## Authors’ contributions

TW, DY, HN, DA and MC contributed to drafting the manuscript. All authors read and approved the final manuscript.
